# Prophylactic effect of *Nigella sativa* against lead acetate induced changes in spermiogram, reproductive hormones and gonadal histology of rats

**DOI:** 10.14202/vetworld.2016.1305-1311

**Published:** 2016-11-27

**Authors:** Mohammed Abdulrazzaq Assi, Mohammed Noor Mohd Hezmee, Yusuf Abba, Md Sabri Md Yusof, Abd Wahid Haron, Mohamed Ali Rajion, Mashaan Abbas Al-Zuhairy

**Affiliations:** 1 Department of Community Health, College of Health and Medical Techniques, Al-Furat Al-Awsat Technical University, Iraq; 2 Department of Veterinary Preclinical Sciences, Faculty of Veterinary Medicine, Universiti Putra Malaysia, 43400, Serdang, Selangor, Malaysia; 3 Department of Veterinary Pathology and Microbiology, Faculty of Veterinary Medicine, Universiti Putra Malaysia, 43400 Serdang, Selangor, Malaysia; 4 Department of Veterinary Clinical Studies, Faculty of Veterinary Medicine, Universiti Putra Malaysia, 43400 Serdang, Selangor, Malaysia; 5 Department of Veterinary Public Health, College of Veterinary Medicine, Baghdad University, Iraq

**Keywords:** histology, lead acetate, infertility, *Nigella sativa*, spermiogram, toxicity

## Abstract

**Aim:**

This study was designed to evaluate the prophylactic effect of *Nigella sativa* (NS) treatment on toxic effects induced by lead acetate (LA) on the reproductive hormones, spermiogram and gonadal histology of rats.

**Materials and Methods:**

A total of 20 Sprague-Dawley rats were divided into four groups of five rats each. Group 1 (negative control [NC]) was the NC and was given distilled water, Group 2 served as the positive control (PC) and was administered 10 mg/kg/day of LA per overall survival (OS), Group 3 (T1) was administered 200 mg/kg/daily of NS per OS for a month, and Group 4 (T2) was pretreated with 200 mg/kg/daily of NS per OS for 1 month, followed by 10 mg/kg/daily of LA alone per OS for another. The rats were euthanized at the end of the experimental period for collection of blood and the right caudal epididymis and testis. Serum was used for determination of reproductive hormones by using radioimmunoassay kits. The epididymal segment was cut and homogenized in phosphate-buffered saline, and the homogenate was used for determination of the spermiogram parameters such as sperm concentration, sperm viability, percentage of live sperm, motility and abnormality. Both the epididymis and testis were fixed in 10% buffered formalin for histological processing.

**Results:**

The sperm concentration, general, and individual motilities were higher (p<0.05) in the NC and T1 animals, while the T2 had intermediate and the PC had lower (p<0.05) values of each parameter. The percentage sperm viability was higher (p<0.05) in the T1 and lower (p<0.05) in the PC group. However, percentage abnormality was lower in T1, comparable in NC and T2, and higher (p<0.05) in PC. Spermatogenic cell population and epididymal sperm reserve (ESR) were optimal in control and pretreated animals, while the PC had lower spermatids and ESR. The concentration of estradiol (EST) was lower (p<0.05) in the PC and T2, while leuteinizing hormone (LH) concentration was lower (p<0.05) in the PC, and comparable (p>0.05) between control and T2. The concentration of follicle-stimulating hormone (FSH) was comparable (p>0.05) in all groups, while testosterone (TS) hormone concentration was lower (p<0.05) in the PC and higher in the control and T1 groups.

**Conclusion:**

This study showed the preventive effects of NS administration against alterations in reproductive hormnes, sperm parameters and gonadal histology caused by LA in rats.

## Introduction

Lead is considered as one of the most hazardous and cumulative environmental pollutants that affect all biological systems through exposure from air, water, and food sources [1]. The effects of lead on tissues and organs have been attributed to the excessive generation of reactive oxygen species (ROS), an issue that was recently given much attention. Sulfhydryl antioxidant production is inhibited by ROS, which damages nucleic acids, inhibits the repair of DNA and inhibits reactions of enzyme, thus enhancing peroxidation of lipids in cellular membranes [2,3]. Lead induced oxidative stress promotes hydrogen peroxide (H_2_O_2_) generation [4]. Lead acetate (LA) has been shown to decrease male reproductive organ function by causing testicular tissue alterations in the histological patterns of the testis [5].

*Nigella sativa* Linn. (NS) is an annual herb that belongs to the family Ranunculaceae and is most extensively investigated for its therapeutic purposes [6,7]. Both the seed and oil of NS have been shown to exhibit different therapeutic properties which include antiparasitic, antifungal, anticancer, antimicrobial, anti-inflammatory, and effects against toxicity [8,9]. The ameliorative effects of NS against sodium valproate [10], heat stress [11], LA [12], isoproterenol [13], tramadol [14], and cisplatin toxicity [15] have been reported in literature.

NS oil was shown to counteract the impairment in epididymal sperm characteristics caused by H_2_O_2_ exposure [16]. Similarly, both the oil and seed of NS have been shown to increase sperm production, semen quality, sperm count, and volume of semen, seminiferous tubular diameter, increased sperm motility and percentage fertility, decreased sperm abnormality index as well as increased hormonal levels [17]. The administration of LA to male rats have also been reported to affect the levels of reproductive hormones; testosterone (TS), estrogen and luteinizing hormone (LH) as well as reduce sperm viability, motility, concentration, gonadal weight and increase sperm abnormalities, and degenerative and necrotic conditions in the testis [5,18-20].

NS is a popular folkloric medicinal plant whose seeds, leaves and oil have been used to treat various sicknesses for centuries. The seed and oil of NS have been reported to be safe with a high safety margin following oral intake. NS have been reported to exhibit potent anti-inflammatory, analgesic, anticarcinogenic, antidiabetic, antiulcer, antimicrobial, and antiparasitic activities [9,21]. In related studies, both administrations of NS seed and oil have been found to improve sperm counts, sperm viability and motility, testicular weights, decrease sperm abnormalities, and testosterone concentration [10,17,22]. Similarly, increased levels of reproductive hormones were observed after administration of NS oil in male rats [23].

Since most of these studies used NS as a therapeutic rather than a prophylactic regiment, this study was hence designed to evaluate the prophylactic effect of NS pretreatment on LA administration with emphasis on sperm production index, reproductive hormonal levels and histology of the testis and epididymis of rats.

## Materials and Methods

### Ethical approval

The animal experimental protocol used in this study was approved by the Institutional Animal Care and Use Committee (IACUC) with reference number: UPM/IACUC/AUP-R047/2015, in accordance with the standard guidelines on usage and care of laboratory animals.

### Preparation of NS and LA solutions

Black seeds (NS) were obtained, cleaned, and grounded using an electric grinder (National Blender 8011S, Model HGB2WTS3, USA) for 10 min to get water-soluble powder. A suspension of 10 g/L of the powder was prepared for this experiment. LA (Oxford Lab., Co., India) was dissolved in distilled water at a concentration of 10 mg/kg body weight (BW) and administered to the rats via a gavage tube.

### Animal grouping and treatment

About 20 adult male rats were randomly divided into four groups of five animals each. Hygienic condition was maintained by changing the bedding weekly. The animals were kept for 15 days for acclimatization before commencement of the experiment.

Group I negative control (NC) was given distilled water, orally. Group II (LA treated group) (positive control [PC]) was given 10 mg/kg of LA, orally for 30 days [24,25]. Group III (NS treated group) was given 200 mg/kg BW of NS water suspension, orally for 30 days [26,27], while Group IV (NS followed by LA treated group) was pre-treated with NS 200 mg/kg BW, orally for 30 days [13], followed by LA 10 mg/kg BW daily for another 30 days [24,25].

### Sperm counts (sperm concentration, viability, individual motility, general motility, and abnormality)

The right caudal epididymis was immediately placed in 2 mL of phosphate-buffered saline and cut into about 200 pieces using a surgical micro-scissor to release the spermatozoa from the epididymal tubules. Epididymal semen suspension (ESS) was immediately incubated at 37°C for further examination. 10 µl of ESS was added to 190 µl of formal saline, to make a dilution factor of 1:20. The total sperm concentration was determined using a Neubauer hemocytometer as described previously by Yokoi *et al*. [28]. To evaluate the mass motility, 5 µl of the suspension was put on a glass slide and examined under a phase-contrast microscope at a magnification of 100×. Mass motility was graded using the percentage motility of the spermatozoa. Moreover, general motility was evaluated by taking 10 µl of ESS on glass slide, covered with cover slip and examined under phase-contrast microscope at 400× magnification. Eosin-nigrosin stain was used to stained the sperm to determine its viability and morphology.

### Processing of tissue samples for histopathology

Tissue samples of the testis and epididymis were collected in 10% neutral buffered formalin and fixed for 48 h. Formalin-fixed tissues sections were processed through serial dehydration in ethanol, embedded in paraffin wax, sectioned at 5-6 µm and stained with hematoxylin and eosin for histopathological examination of each animal through light microscopy at 200× and 400× magnification. Photomicrographs of microscopic focal fields were taken using Miotic^®^ microscope

### Radioimmunoassay for follicle stimulating hormone (FSH), LH, estradiol (ESR) and testosterone measurements

Beckman Coulter Immunotech Radioimm unoassay kits were used for the plasma determination of FSH, LH, estradiol and testosterone concentrations as previously described [29]. Briefly, 50 µl of the calibrator and plasma from different groups were added to 500 µl of tracer in an anti-LH or anti-FSH or anti-estradiol antibody-coated tubes, mixed and incubated at 18-25°C in a shaker set at 350 rpm. Wallac Wizard Gamma Counter model 1470 was used to determine the counts per minute (cpm) of the solution. For the evaluation of testosterone concentration, 200 µl of the plasma was mixed 2 ml of ethyl ether and vortexed vigorously for 1 min. The mixture was kept at −18°C until a frozen aqueous phase was achieved. The aqueous phase was separately collected and in a water bath at 37°C until evaporation. The resultant dry ether extract was re-dissolved in 200 µl of recovery buffer, vortexed vigorously, and mixed in tracer antibody-coated tubes. The tubes were incubated at 37°C water bath for 1 h, and the solution was used for determination of total cpm using Wallac Wizard Gamma Counter model 1470 [29].

### Statistical analysis

Data obtained from the spermiogram was summarized as mean±standard error and analyzed with Graph Pad Prism 6.0 using one-way analysis of variance with Tukey multiple comparison *post-hoc* test. A value of p<0.05 was considered significant.

## Results

### Changes in spermiogram parameters

**Figure-1:**
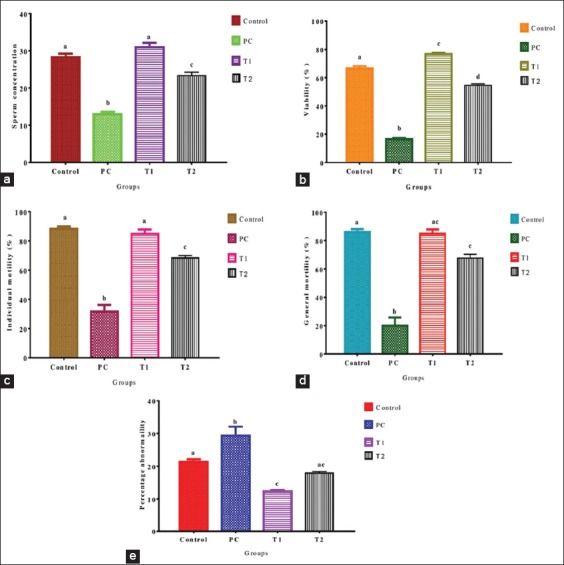
Sperm concentration (a) percentage viability, (b) individual motility, (c) general motility, (d) percentage abnormality, (e) in different experimental groups. a,b,c,d Bars with different superscript indicate statistical significance at p<0.05.

In this study, it was observed that LA had a deleterious effect on the spermiogram of rats. Consequently, prior administration of NS was able to prevent the development of such effects. Sperm concentration and individual motility were both higher (p<0.05) and comparable in the NC and T1 groups, while the PC had a lower (p<0.05) value compared to all groups (Figure-1a and c). The percentage viability was different (p<0.05) in all groups, with the T1 having a higher value and the PC with a lower value (Figure-1b). The general motility was higher (p<0.05) and comparable in the NC and T1 groups; however, there is no difference (p>0.05) between the T1 and T2, while the PC had a lower (p<0.05) general motility (Figure-1d). The percentage abnormality was higher (p<0.05) in the PC and comparable (p>0.05) between NC and T2, and T1 and T2 (Figure-1e).

### Histological findings

**Figure-2:**
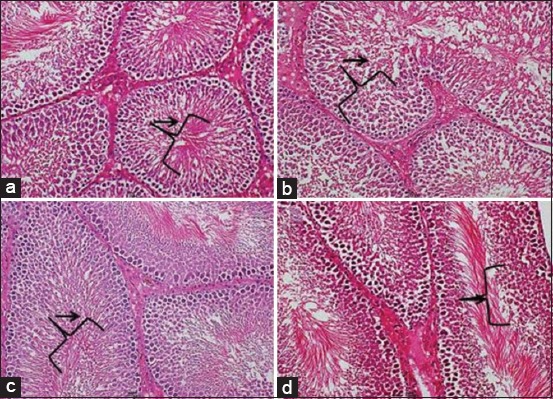
Photomicrograph section of the testis of rats from (a) control group, showing normal spermatogenesis characterized by different spermatogenic cells within the lumen of the seminiferous tubules (arrow), (b) positive control group, showing spermatogenic cells and evidence of spermatogenesis depicted by reduced number of sperm maturation stages in the seminiferous tubular lumen (arrow), (c) T1 group, showing optimal spermatogenesis with presence of all sperm maturation stages (arrow), (d) T2 group, showing normal distribution of spermatogenic cells with the seminiferous tubules (arrow) (H and E, 200×).

Optimal spermatogenesis was seen in the testis of NC and T1 animals. The PC group had vacuolations within the seminiferous tubules, as well as low distribution of spermatogenic cells. In the T2 group, spermatogenesis was optimal in some tubules (70%) and moderate in others (30%). Tubules with moderate spermatogenesis had low distribution of spermatogenic cells. The distribution of sertoli cells and spermatogonia was not different among all groups (Figure-2).

**Figure-3:**
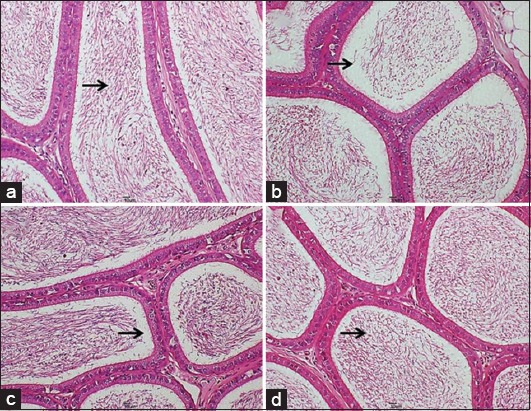
Photomicrograph section of the epididymal tail from (a) Control group, showing optimal epididymal sperm reserve (ESR) of 80-90% (black arrow), (b) positive control group, showing reduced ESR of 60-70% with sparse distribution of sperms (black arrow), (c) T1 group, showing marked distribution of sperms appearing as dense fibers within the lumen (black arrow), (d) T2 group, showing good ESR with moderate sperms distribution (black arrow) (H and E, 200×).

The epididymal sperm reserve (ESR) is an estimate of the epididymal tail luminal filling with spermatozoa. It indicates the state of sperm production as either optimal (80-100%), moderate (>50%), or low (<50%). The NC, T1 and T2 had optimal sperm reserves within the tail of the epididymis. However, the distribution of sperm cells was dense in the T1 group when compared to other groups, as evidenced by wave like pattern. The PC group had a reduced ESR depicted by moderate distribution of sperm cells in the epididymal tail (Figure-3).

### Reproductive hormonal concentrations

**Figure-4:**
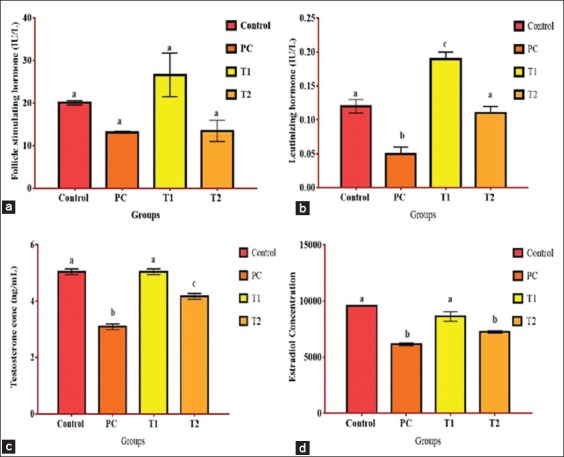
Reproductive hormonal concentration showing the difference in (a) Follicle-stimulating hormone (b), luteinizing hormone, (c) testosterone hormone and (d) estradiol in rats following pretreatment with Nigella sativa and lead acetate exposure. a,b,c Bars with different superscript indicate statistical significance at p<0.05.

There was no significant difference in the concentration of FSH in all groups. However, the concentration of LH was significantly lower in the PC group, while the T1 group had a higher value that was elevated above the control and T2 groups. The concentration of TS was significantly lower in the PC once compared with other groups. Similarly, EST concentration was also lower in the PC and T2 groups and higher in the control and T1 groups (Figure-4).

## Discussion

In this study, we used the NS seeds to evaluate its ameliorative effects against the toxicity of LA. The results showed that NS had a protective effect in the reproductive organs after the inducement of LA toxicity. However, the optimal dose of NS at 200 mg/kg BW reduced the harmful effects that LA caused in these organs.

There are widely documented deleterious effects of LA on the developing and mature male reproductive organs and fertility in previous studies. Lead is known to induce malfunctioning of the reproductive organs by directly affecting its morphological structure, thereby also causing decreased sperm quality [30,31]. The ameliorative effects of NS against several toxic compounds have been reported in literature [10,13-15,32]. Al-Masri [33] had reported that LA caused significant decreases in sperm motility, count and viability of sperm, while there was in an increase in sperm abnormality. Similarly, Chowdhury [34] also reported the toxicity of lead in the male reproductive system by inducing alterations in sperm morphology, count, motility as well as a decrease in the male sexual hormones. Since the major function of testes is spermatogenesis and hormone production, damage to the testicular tissue by the toxic effects of lead impairs both the spermatogenic process and hormone production [35,36]. However, several studies in other rat strains and other rodents indicate fairly consistently that blood Pb concentrations >30-40 µg/dL during at least 30 days of administration was associated with impairment of spermatogenesis and reduced concentrations of circulating androgens. This concurs with the findings of this study where it was observed that the LA (PC) group had reduced fertility index (sperm concentration, percentage viability, individual motility, general motility) and increased percentage abnormality when compared with the NS pre-treatment and control groups. Similarly, the PC group had less number of spermatids and vacuolations in the seminiferous tubule with reduced ESRs.

In a related study, a significant increase in the wall thickness of testicular seminiferous tubules was observed in mice administered 0.3 mL of the crude oil of NS [37]. Significant increases in BW gain, reproductive parameters (seminiferous tubules thickness and diameters, account of spermatogonia, primary and secondary spermatocytes, spermatids, free spermatozoa, account of Sertoli and Leydig cells, diameter of Leydig cells and the height of epithelial cells entirely covered epididymal caudal) and hormones (testosterone and FSH) were observed with treatment of alcoholic extract of black seed [38]. In this study, NS pretreatment was observed to significantly improve the fertility indexes by improving sperm concentration, percentage viability, individual motility, general motility and decreasing percentage abnormality. Furthermore, there was enhanced spermatogenesis and ESRs in pre-treated rats exposed to LA.

Furthermore, the administration of different doses of LA was shown to cause reduced testicular weight, sperm concentration and motility, increased sperm abnormalities and increased testicular degenerative changes in previous studies by Elgawish and Abdelrazek [5], Makhlouf *et al*. [18], and Allouche *et al*. [19]. Although there are numerous reported literatures on the ameliorative effect of NS oil against toxic injuries to the male reproductive organs and hormonal level decreases induced by other compounds [10,11,15], there appears to be very little information on the effect of both its oil and seeds on LA induced testicular injuries and hormonal deficits in male animals.

The administration of NS to normal rats has been shown to boost the sperm production profiles and TS levels, indicating that the compound enhances male fertility [17,23]. In this study, LA reduced the concentration of sperm at the maturation stage in the testis and reduced tail ESRs indicating suppression of spermatogenesis, however, rats treated with graded doses of NS showed remarkable improvements in both spermatogenesis and ESRs. It is, therefore, obvious that NS prevented testicular tissue damage and even enhanced sperm production at 200 mg/kg.

The reproductive hormones TS, EST and LH are important component of male sexual development and fertility. A significant decline in TS or an increase in EST and LH have been shown to adversely affect sexual maturity and fertility in male animals [39]. In this study, decreases in estradiol were observed in the PC and T2 groups on days 30 and 60, respectively, while LH was decreased in PC and comparable between control and T2. The FSH concentration was comparable in all group. This showed deleterious effects of LA on the PC group and suppression of FSH production in the high dose treatment group (NS 200 mg/kg). The decline in the level of TS in the PC in comparison to the the control and T1 groups can be attributed to the lesions observed at histopathology; this ultimately resulted in low sperm in both maturation stage and epididymal reserves which were translated into low sperm concentration and viability. Based on our recent study investigating the therapeutic effect of NS treatment against chronic LA induced toxicity, we observed that concurrent administration of graded doses of NS with LA for 90 days resulted in amelioration of the toxic effects of LA on the gonadal histology, spermiogram, reproductive hormones and oxidative stress enzymes. The study concluded that NS especially at a dose of 200 mg/kg BW had enhanced therapeutic effects against chronic LA induced toxicity in rats [40].

The administration of NS oil to normal male rats was reported to increase the levels of TS, LH and FSH after 30-60 days, indicating a positive effect on the hormonal production [17,22,23]. Based on these reports, we can say that the NS seed powder played a therapeutic role on the testicular tissue and Leydig cells, hence maintaining TS production at normal levels throughout the experimental course. This agrees with a previous study, where NS oil at 250 mg/kg was reported to increase the level of TS in rats exposed to sodium valproate [10].

## Conclusion

This study has shown the promising effect of NS pre-treatment in LA toxicity in male rats. The results showed enhanced fertility indexes in NS pre-treated rats, thus showing the important role of NS in preventing infertility in male rats exposed to LA.

## Authors’ Contributions

All authors mentioned have made substantial contribution to this manuscript and approved the list of authors in this manuscript. The authors: MAA, MNMH, YA, MSMY, AWH, MAR and MAAZ participated in the conception and design. MAA drafted the manuscript, while MHMN, YA, MSMY, AWH, MAR and MAAZ each critically revised and contributed to the intellectual content, and also editing of the paper. They all approved the final draft for submission and the list of authors.
